# Violin Varnishes: Microstructure and Nanomechanical Analysis

**DOI:** 10.3390/molecules27196378

**Published:** 2022-09-27

**Authors:** Marianne Odlyha, Jeannette J. Lucejko, Anna Lluveras-Tenorio, Francesca di Girolamo, Stephen Hudziak, Adam Strange, Alexandra Bridarolli, Laurent Bozec, Maria Perla Colombini

**Affiliations:** 1 Department of Biological Sciences, Birkbeck, University of London, London WC1E 7HX, UK; 2 Department of Chemistry and Industrial Chemistry, University of Pisa, Via Giuseppe Moruzzi 13, 56127 Pisa, Italy; 3 Department of Electronic and Electrical Engineering, University College London, London WC1E 6BT, UK; 4 Eastman Dental Institute, University College London, London WC1E 6DG, UK; 5 Getty Conservation Institute, 1200 Getty Center Drive, Suite 700, Los Angeles, CA 90049, USA; 6 Faculty of Dentistry, University of Toronto, Toronto, ON M5G 1G6, Canada

**Keywords:** in situ imaging, AFM, SR micro-FTIR, violin, nanomechanics

## Abstract

The aim of the current work is twofold: to demonstrate the application of in situ non-invasive imaging by portable atomic force microscopy (AFM) on the surfaces of a violin and to integrate compositional and mechanical analysis at the nano scale level on model samples of varnished wood. These samples were prepared according to traditional recipes by an Italian lute-maker family well practised in the art. Samples of oil and spirit-based varnishes on maple wood, naturally and accelerated light aged, were studied. AFM was used to measure the nanomechanical properties of the model samples and established that the spirit-based varnish was stiffer than the oil-based. Synchrotron radiation micro- Fourier Transform Infra-red analysis of the layer structure revealed that stiffer spirit-based varnish showed less penetration into the wood than the oil-based. Further PeakForce Quantitative Nanomechanical Mapping (QNM) demonstrated a difference in adhesion values between the oil- and spirit-based samples.

## 1. Introduction

To date, in the conservation of musical instruments, it has been a challenge to understand the effect of the varnishing process on wood and whether this process affects the overall performance of varnished wooden instruments. A recent paper by Gilani showed that laboratory varnishes with various compositions penetrated differently into the wood structure [1] as recorded by non-destructive methods such as vibration tests and X-ray tomography. A comprehensive review has also been made of the chemical composition and multi-layered structure of varnishes of historical musical instruments, in particular stringed instruments of the violin family from the 17th and 18th centuries [2]. In the current paper, the information on depth penetration of varnishes of different chemical composition is combined with their mechanical properties measured at the nano-scale. This follows on from previous studies where attention was directed to the physicochemical state of varnish and how this affects the mechanical response of wood [3,4,5]. It was observed that the stiffness of the wood measured in tension in the radial direction increased in the presence of the dammar varnish layer and these results were consistent with what was reported previously using a theoretical approach on spruce plates with natural resins [6].There was also difference in the mechanical properties and response to relative humidity (RH) of varnished wood panels (pine) stored within protected enclosures such as micro-climate frames and showcases and outside these enclosures together with a difference in the surface topography of the varnished surfaces. It has recently been shown that even preparatory treatments affect the properties of the wood and certainly influence subsequent penetration of the varnish [7].

Several papers reported analytical study on varnishes [4,8,9,10]. For example studies on historical stringed musical instruments from the collection of the Vincenzo Bellini Conservatory in Palermo (Italy) showed that the varnish on all the sampled instruments has a consistent formulation containing a mixture of a diterpenoid resin, shellac and a drying oil [11]. Chemical analysis at the molecular level using gas chromatography and mass spectrometry (GC/MS) showed differences in the chemical composition of varnished panels maintained within and outside the enclosures [12]. Recently, the composition of the materials and the chemical composition of each varnish layer was reported by using spatially resolved synchrotron radiation micro-FTIR spectroscopy (SR-FTIR), which included studies on early 18th century musical instruments [13], a late 16th cent. Venetian lute [14] and instruments which were undisputed representative examples of Stradivari’s work [15].

An important consideration in the evaluation of the state of preservation of a string instrument is the application of non-invasive and non-destructive techniques. For this reason, optical coherence tomography (OCT) [16] has been applied. A recent in situ study of the surface of an 18th century Italian violin provided important information for its conservation treatment and demonstrated that it was possible to characterize the thickness of varnish layers and image the three-dimensional wood structures below the varnishes [17]. The relevance of the mechanical properties of varnishes has recently been highlighted [3]. To achieve a non-invasive non-destructive approach, the use of force spectroscopy with Atomic Force Microscopy (AFM) could be considered, and, in this paper, a preliminary study on model samples is included (Table 1, described in Section 4.1.2). Force spectroscopy with AFM has emerged as a powerful technique as it is able to provide information on the nanomechanical properties of a wide variety of materials at the nanometre/nanonewton scale [18]. At this stage, in situ AFM imaging has been achieved on 18th cent parchment. It was possible to detect changes in the collagen in parchment, which can cause surface cracking resulting in loss of text and can increase the vulnerability of parchment to aqueous cleaning agents [19]. In the literature, there are few studies using AFM to evaluate the surface properties of woods. An early report by Vlad-Cristea et al. [20] used both nanoindentation and atomic force microscopy to investigate whether the exterior durability of waterborne coatings improved with inorganic nanosized UV-absorbers. In their study, AFM was used to detect the very early stages of surface roughening and blistering that occurs during weathering. The recent research by Mao et al. [21] presents an accurate atomic force microscopy (AFM) method of investigating wood aging. The team’s work demonstrated a novel AFM-based method of analysing wood aging. The technique was able to characterize changes to the wood surface by measuring the relative adhesion force between the wood sample and the AFM tips. It was concluded that the structures most suitable for monitoring aging at early stages were the wood pits. Others have used AFM to characterize the entire wood cell wall mechanically at the nanometre level [22,23]. Recently, Czibula et al. [24] demonstrated a decreasing trend in the longitudinal and transverse viscoelastic properties of wood pulp fibers at different relative humidity. However, they hypothesised that the unknown microfibril angle (MFA) and the anisotropy of the material could have an influence on the results.

The current work is aimed at evaluating the feasibility of in situ imaging by using Atomic Force Microscopy (AFM) not only on reference systems simulating the stratigraphy of a violin but also on a historical violin in order to obtain information on the preservation of the varnished surface. In situ AFM does not require sampling as the instrument is fully portable and would allow for in situ examination of the varnish surface at the nanoscale level. To complement surface information, chemical analysis using micro-Fourier Transform Infra-red analysis has also been performed. Actually, Infra-red specular reflection and diffuse-transflection techniques have been reported as a method for in situ non-destructive analyses of varnishes on historical violins with a view to indicate state of degradation of varnishes [25,26]. A recent paper on modified asphalt binders [27] and on bamboo fibre cell walls and their composites has demonstrated the value of these additional data as basic nanomechanistic properties, adhesion, energy dissipation, and deformation of the modified asphalt binders were determined [28].

This paper reports the results obtained by in situ AFM imaging on a historical violin bought in 1946 from Boosey & Co in London and Synchrotron radiation micro-FTIR spectroscopy (SR-FTIR) on model system samples (Table 1, described in Section 4.1.2) prepared by a violin maker (Gabriele Carletti who continued the family tradition of constructing and restoring string instruments) according to traditional recipes. In addition, force spectroscopy with AFM and peakForce QNM mode of AFM were used to measure the nano mechanical properties of model violin samples and to provide information on adhesion values of the surfaces.

## 2. Results and Discussion

**Figure 1:**
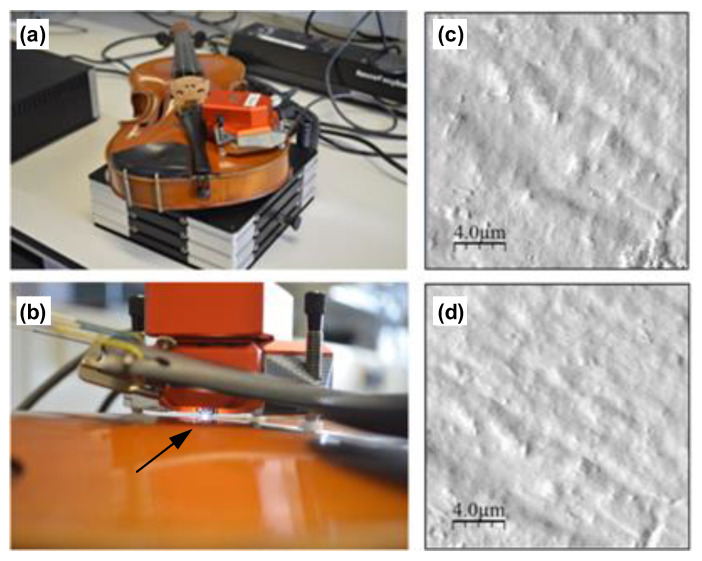
(**a**) Nanosurf Easyscan 2 AFM head on a violin placed on an anti-vibration table (**b**) closer view of AFM head on the violin placed on plastic spacers to protect the surface of the violin and showing location of the AFM tip (black arrow) (**c**,**d**) AFM images (20 μm × 20 μm) recorded directly on the violin surface in two different locations [location (**c**) z-scale image = 1.28 μm/Roughness Ra 0.176 μm; location (**d**) z-scale image = 2.03 μm/Roughness Ra 0.291 μm].

Atomic force microscopy (AFM) was applied to violin surface analysis. The AFM head was placed directly on the instrument as shown in Figure 1a, and the whole violin was placed on an anti-vibration table. Figure 1b shows the tip in contact with the surface. To the right side in Figure 1b it is possible to see one of the legs resting on a plastic spacer used to protect the surface of the violin on which the AFM head rested. It was found that operation in intermittent contact mode provided better images. Figure 1c,d show the resulting AFM images taken in different locations on the surface of the violin. The surface shows the directional nature of varnish application following the direction of the grain of the wood, some small deformations, and some particulate matter. This is the first time that it has been demonstrated that it is possible to image directly the surface of a violin using AFM. The ability to visualise the surface at the nanoscale level provides information on the state of preservation of the varnish and may indicate the type of preparation used. AFM has been previously used in studies on violins, in particular in the study of structural changes in cuticles on violin bow hair caused by wear and in comparison of synthetic hair and natural horse tail as used in professional orchestras [29,30].

**Figure 2:**
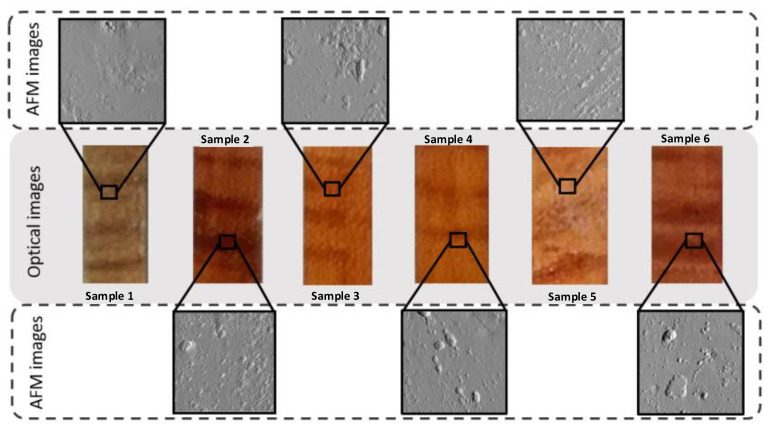
Model systems optical images together with AFM images of samples (1 to 6) before ageing: oil-based (1 and 5); AFM image 1, z scale image = 159.4 nm/Roughness 19.1 nm; AFM image 5, z scale image = 164.5 nm/Roughness 18.2 nm; and spirit-based varnish layers (2, 3, 4, 6); AFM image 2 z scale image = 235.6 nm/Roughness 10.5 nm; AFM image 3, z scale image = 522.6 nm/Roughness 33.6 nm; AFM image 4, z scale image = 823.9 nm/Roughness 18.6 nm; AFM image 6, z scale image = 260.0 nm/Roughness 14.5 nm on top of maple wood over preparation layers of various compositions (Table 1, described in Section 4.1.2).

The second aim of this study was to evaluate the mechanical properties of the varnished surfaces at the nanoscale level. For these measurements, a set of model varnish samples was prepared according to traditional practices for violins. Table 1, described in Section 4.1.2 shows the recipes used in the preparations. AFM has long been recognised as a useful instrument for the evaluation of local mechanical properties of materials. A collection of force-distance curves at each pixel allows for mapping of the elastic properties [31]. Recent advances in Force spectroscopy with AFM have meant that additional material properties such as dissipation, adhesion, and deformation are mapped simultaneously with topography at nanoscale resolution [18,32]. Figure 2 shows corresponding AFM images (10 μm × 10 μm) of the model samples. Several locations were tested. Naturally aged samples (4 years) and accelerated aged samples (2 weeks of Solar box) were studied.

**Figure 3:**
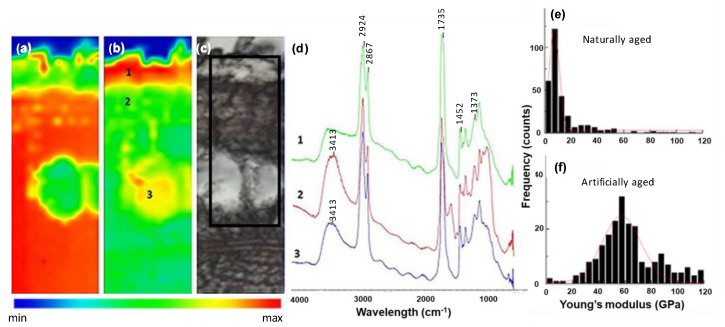
(**a**–**d**) shows SR-micro-FTIR mapping for the naturally aged oil-based varnish (Figure 2 sample 1): chemical image mapping of the absorbance at (**a**) 1000 cm^−1^ and (**b**) 1735 cm^−1^, respectively, and (**c**) photomicrograph of the microtomed cross-section of width 10 μm. (the squared area outlined in black has a depth of 250 μm and indicates depth of penetration of the oil into the wood); and (**d**) FTIR spectra of layers (1, 2, 3) as shown in (**b**) and corresponding to varnish, penetration areas of oil in wood, and oil, indicating high penetration into the wood porous structure (around 200 µm). (**e**,**f**) shows mechanical data and distribution of Young’s modulus in the unaged which, at time of analysis, had undergone four years of natural ageing in the dark (**e**) and the artificially accelerated aged sample (**f**) where median values of Young’s modulus are (7.04 +/− 1.75) GPa and (57.2 +/− 5.1) GPa, respectively.

**Figure 4:**
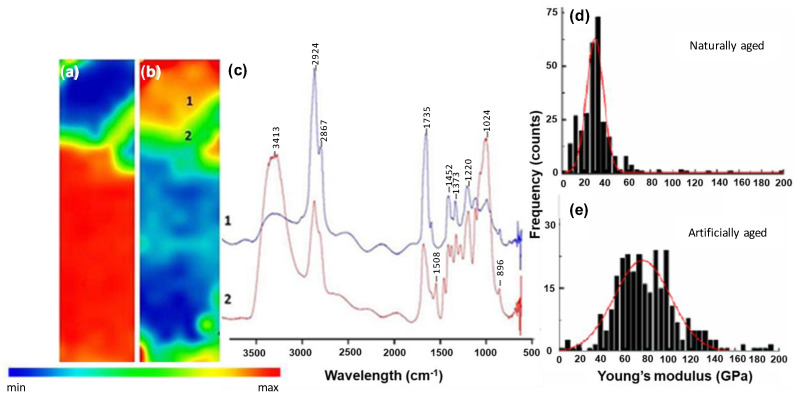
(**a**–**c**) shows SR-micro-FTIR mapping of the naturally aged spirit-based varnish (Figure 2 sample 6): chemical image mapping the absorbance at 1000 cm^−1^ (**a**) and 1700 cm^−1^ (**b**) respectively. FTIR spectra (**c**) representative of the varnish layer (1-blue line) and the underlying wood (2-red line) in (**b**). The spirit-based varnish remains on the surface with a clear separation from the wood as shown by the difference in spectra for layers 1 and 2. (**d**,**e**) shows mechanical data and distribution of Young’s modulus in naturally aged (4 years) (**d**) and artificially accelerated aged (**e**) samples where median values of Young’s modulus are 29.7 +/− 3.1 GPa and 75.7 +/− 9.1 GPa, respectively.

With regard to the mechanical properties of these samples measured at the nanoscale level, Young’s modulus values were calculated from the measured force distance curves obtained from AFM. This information was complemented with chemical analysis of the layers in the varnished wood samples’ cross-sections using synchrotron radiation micro-Fourier Transform Infra-red analysis. Figure 3 and Figure 4 show the spectral and the mechanical data for both naturally aged and accelerated aged oil based varnish sample 1 (Figure 2), and spirit-based varnish sample 6 (Figure 2).

Oil-based varnish shows a characteristic C=O stretching vibration centred at 1735 cm^−1^ with a shoulder at 1708 cm^−1^, characteristic of the ester linkages of the triglycerides from the drying oil and the free fatty acids formed during its ageing. FTIR spectra of the varnish layers of the oil-based sample in Figure 3d showed typical bands for oil-based varnish (i.e., the strong and broad carbonyl stretching absorption and some medium intensity absorption bands at 1452 cm^−1^, 1373 cm^−1^ (from the resin component also present) and accompanying alkyl stretching bands). Strong peaks with two maxima at 2924 cm^−1^ and 2867 cm^−1^ are due to aliphatic C-H stretch of alkyl groups. The numbers (1 to 3) in Figure 3b,d refer to the different composition areas: 1 contains oil varnish, 2 contains wood and oil varnish and 3 contains oil varnish on the layer of wood support, being the result of the penetration of the varnish.

The FTIR data for the spirit-based sample (Figure 4c) show a C=O stretching band centred at 1696 cm^−1^ and two medium intensity absorption bands at 1452 cm^−1^, 1373 cm^−1^ which highlight the presence of a terpenoid resin [13]. These spectra also show strong peaks with two maxima at 2924 cm^−1^ and 2867 cm^−1^ corresponding to aliphatic C-H stretch of alkyl groups, which are more intense in the varnish layer (blue line) than in the wood (red line). Chemical maps of the spirit based model show that varnish remains on the surface with a clear separation from the wood as shown by the difference in spectra for layers 1 and 2 (Figure 4c).

The FT-IR spectra of the wood layer in both Figure 3 and Figure 4 show a broadening to lower wavenumbers of the band at 3413 cm^−1^. This may be caused by the residual water present in the wood layer due to the fact that the wood is a highly hygroscopic material but also due to O-H stretching vibration from alcohols (3600–3300 cm^−1^) and carboxylic acids (3300–2500 cm^−1^), present either in polysaccharides or lignin fractions of wood [33,34]. The broad band between 900 and 1100 cm^−1^ in Figure 4c (red line) contains many signals from cellulose vibrational modes, in particular, the band at 1024 cm^−1^ related to the C-O bond. At 896 cm^−1^ the typical signal for C-H stretching involving C1 carbons in the glucose ring is visible. The absorption band at 1508 cm^−1^, attributed to aromatic skeletal vibrations in lignin as well as the band at 1220 cm^−1^ [33,35,36] is also present.

Figure 3 and Figure 4a,b show the chemical images at 1000 cm^−1^ and 1700 cm^−1^ and the distribution of the wood support and the varnish, respectively. From the FTIR maps, it is possible to observe the degrees of penetration of the vanish layers into the wood support. The oil-based varnish shows a high penetration into the wood porous structure (around 200 µm) (Figure 3d) while the spirit based one remains on the surface with a clear separation from the wood support (Figure 4c).

Figure 3d,e shows the distribution of Young’s modulus of the varnished wood oil-based sample. In the accelerated aged sample, there is a clear shift in the maximum of the distribution of Young’s modulus to higher values, i.e., 60 GPa (Figure 3f). There is also a broader distribution in the aged sample. The maximum value of Young’s modulus for the unaged sample, which had been naturally aged (4 years) at the time of analysis, lies in the range 5–10 GPa, and this correlates with values found for maple (hardwood) (elastic modulus 12.62 GPa) [37].

Figure 4d,e shows the distribution of Young’s modulus on the varnished wood spirit-based sample, sample 6. In the unaged sample (Figure 4d), which had also been naturally aged (4 years) at the time of analysis, the maximum value for the modulus is higher than in the oil-based sample and is in the region 30–40 GPa. In the accelerated aged spirit-based sample, there is also a shift in the maximum of the distribution of Young’s modulus to higher values but the distribution of the aged sample (60–100 GPa) is broader (Figure 4e) than in the oil-based sample.

**Figure 5:**
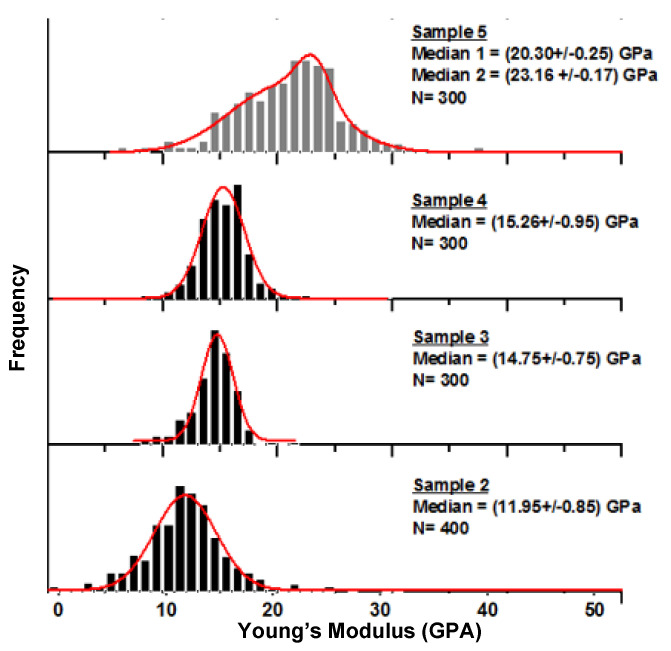
Mechanical data and distribution of Young’s modulus obtained for sample 2, 3, 4 and 5.

Nanomechanical measurements were also performed on samples 2, 3, 4 and 5 (Figure 5). The spirit-based samples (black bars) show similar values of modulus to that of sample 6. The second oil-based sample (sample 5 grey bars) has a higher modulus value, unlike that of the other oil-based sample (sample 1), and also differs from sample 6 as seen in the adhesion data below.

**Figure 6:**
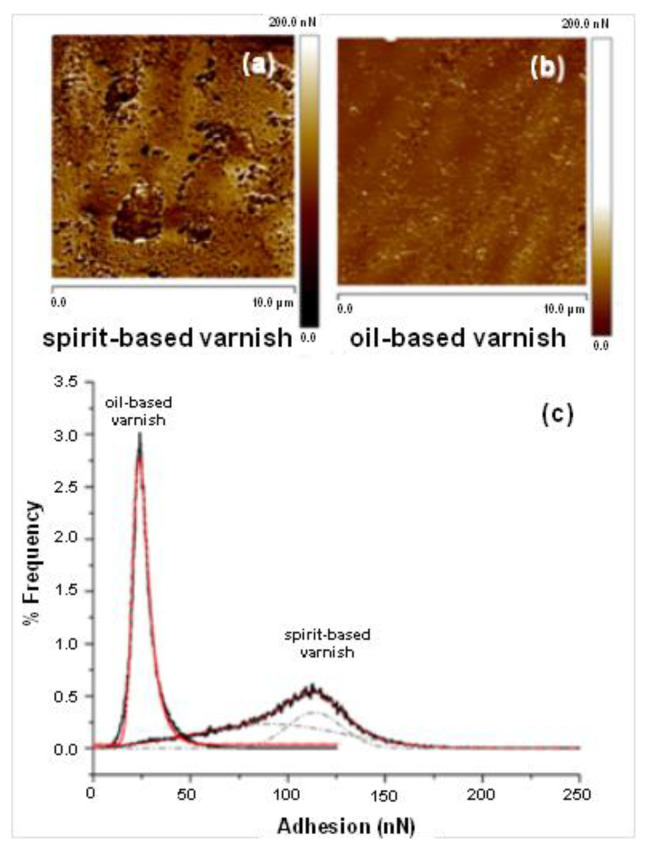
The PeakForce adhesion image of the naturally aged spirit-based sample (sample 6) is shown in (**a**) and the image for naturally aged oil-based varnish sample is shown in (**b**). Bearing analysis gave large differences in the distribution of adhesion values for the two samples (**c**): the oil-based varnish has a narrow range of adhesion values while the spirit-based has a broad distribution and can be fitted to 2 curves, indicating the possibility of greater heterogeneity in the mechanical properties of the surface of the spirit-based varnish.

Information on adhesion values of the surfaces of the model violin samples was also obtained. The PeakForce Adhesion image of the naturally aged spirit-based sample (sample 6, Figure 2) is shown in Figure 6a, and the image for the naturally aged oil-based varnish (sample 5, Figure 2) is shown in Figure 6b. These were recorded from PeakForce QNM measurements. Bearing analysis showed that the two samples gave large differences in the distribution of adhesion values, Figure 6c. The spirit-based sample has a higher maximum adhesion value than the oil-based sample with a broader distribution of values. The oil-based sample (sample 1, Figure 2) has a lower maximum adhesion value and a narrower range of values. Figure 6b shows that the distribution of adhesion values for the spirit-based varnish can be resolved into two contributions to the overall peak, corresponding to regions of lower and higher adhesion values as seen in the image in Figure 6a. This could be a future marker that can be used to characterise behaviour of spirit-based varnishes. Further work is in progress.

## 3. Conclusions

In this study it has been demonstrated for the first time that it is possible to obtain AFM images in situ on the surface of a violin using the portable EasyScan 2 AFM instrument. This opens possibilities for imaging on historical violins in collections and for assessment at the nanoscale of the state of preservation of the varnish. Peak force tapping AFM with quantitative nanomechanical analysis provides a tool for assessment of nanomechanical properties of multiphase materials. AFM has provided information on the nano-mechanics of oil-based and spirit-based varnishes and the changes that occur on accelerated ageing. Peak Force AFM has provided additional information on adhesion values. Synchrotron radiation micro-FTIR spectroscopy (SR-FTIR) has provided information on differences in the chemical composition of the varnish layers over the wood and in the depth of penetration of the two varnishes. Examination of the layer structure reveals that the stiffer spirit-based varnish showed less penetration into the wood than the oil-based varnish. This is in accord with observations made by X-ray tomography [1,38]. Information on the extent of penetration into the wood and the change in physico-chemical and mechanical properties with ageing is essential as these properties will contribute to the vibro-acoustical properties of the coated wood as indicated in other papers [1,39,40]. The AFM and nanomechanical studies open up a whole new area of investigation of varnished musical instruments and will provide essential data. Though it has been recognised that the impact of vanish can affect the final sound quality of violins via changes in mechanical properties, there are fewer measurements of, for example, changes in stiffness of varnishes from historical instruments and fewer studies made than of the chemical composition of varnishes from historical instruments [41]. This is mainly due to the invasive nature of mechanical tests generally used. In our study we have shown the potential of non-invasive imaging and the possibility of future non-invasive mechanical measurements on historical instruments.

## 4. Materials and Methods

### 4.1. Samples

#### 4.1.1. Violin Instrument

The violin which was made available for this study shown in Figure 1a,b had been played for about 40 years. It was bought in 1946 from Boosey & Hawkes (Boosey & Hawkes Music Publishers Limited, Aldwych House, London, UK,), and, thus, is likely to be no more than 70 years old. It has never been re-varnished but has been polished with Hidersine 3V violin wax and varnish cleaner (Barnes & Mullins Ltd., Morda, UK).

#### 4.1.2. Model System Samples

For this project, luthier Gabriele Carletti prepared samples using traditional violin varnish recipes. These model systems, shown in Figure 2 and described in Table 1, consist of oil or spirit-based varnish layers on top of maple wood (6 cm × 1.2 cm × 2 cm) over preparation layers of various compositions: water soluble potassium dichromate (also with sodium bicarbonate), cherry gum, gamboge and saffron and then those that are soluble in ethanol and contain dragon’s blood and sandalwood. The varnish layers were (a) oil-based containing stand oil, spirit of turpentine, Venice turpentine, Mastic and Sandarac and (b) spirit-based containing ethyl alcohol, Venice of turpentine, Mastic, Elemi, and Shellac (Table 1). All products were Kremer-pigmente GmbH & Co., Aichstetten, Germany.

### 4.2. Atomic Force Microscopy (AFM)

The portable Nanosurf Easyscan 2 AFM operating in intermittent contact mode was used for in situ imaging of the surface of a violin instrument.

### 4.3. Nanomechanical Measurements

The nanomechanical measurements were performed on the naturally aged samples (4 years after their preparation), which had been stored in the dark, and on the accelerated aged samples.

#### 4.3.1. Young’s Modulus Measurement

A Nanowizard 1 AFM (JPK, Bruker, Ettlingen, Germany) was used to measure the Young’s Modulus of the model violin samples using a standard force-distance curve approach. The cantilever used for the measurements had a spring stiffness of k = 38 N/m (RTESPA, Bruker, Camarillo, CA, USA) with a setpoint of 500 nN. Samples were adhered to a glass slide using double-sided tape, with the varnish side exposed. Force-distance curves (300) were obtained across three separate areas of 50 µm^2^ using a Nanowizard (JPK, Berlin, Germany) in contact mode in the air. All FD curves were processed with the Hertzian Model on the proprietary JPK analysis RampDesigner™ software (Budapest, Hungary).

#### 4.3.2. Adhesion Measurement

A Bruker Dimension Icon with PeakForce QNM was used to perform adhesion measurements on the model violin samples. The probe used for the measurements had a spring stiffness of k = 6 N/m (TAP 150A, BudgetSensors, Izgrev, Sofia, Bulgaria) with a setpoint of 10 nN. QNM adhesion images of 10 × 10 um^2^ were obtained at 5 locations across each of the samples. Using the QNM software (open-source Python package), we measured the maximum adhesion for each pixel in the recorded images and pooled the data to plot the Frequency distribution of adhesion for both the oil-based and spirit-based varnish samples.

### 4.4. Micro-FTIR Measurements

Synchrotron radiation Micro-Fourier Transform Infra-red Analysis (Micro-FTIR) was carried out at the IRIS beamline (BESSY II) at Helmholtz-Zentrum, Berlin (Germany) on a Thermo Nicolet Continuum™ microscope equipped with a mercury–cadmium–telluride (MCT) detector. The sample was mounted on a diamond cell on a motorized microscope stage and raster scanned through the synchrotron beam with a diameter of 15 µm collecting a grid-like pattern of IR spectra spaced in 10 μm increments. Measurements were performed in transmission mode at a magnification ×32 using confocal objectives. Infrared spectra were registered between 4000 and 650 cm^−1^ with a spectral resolution of 4 cm^−1^. An accumulation of 128 scans per point was used. Background spectra were collected under identical conditions with only the BaF_2_ window on which the sections were placed. The spectrum and mapping acquisition was performed by using the OMNIC Atlμs™ software (Waltham, MA, USA) [42]. The sample preparation protocol consisted of the microtoming at 10 µm of the samples with an embedding-free approach as described in the literature [43].

### 4.5. Accelerated Aging

Accelerated aging was performed for two weeks after 10 months’ natural aging in a Solarbox 1500e RH (Erichsen Cofomegra Instruments, Milan, Italy) purchased from Erichsen (Hemer, Germany). Ageing conditions were as follows: 20 °C, 50%RH, excitation with Xenon lamp (wavelength 280–400 nm) power 400 W. A soda-lime glass UV filter was used to simulate indoor exposure. Irradiation uniformity was guaranteed by a parabolic reflector chamber with the Xenon lamp in focus.

## Figures and Tables

**Table 1:** Preparation layer composition of a set of varnished wood samples (1 to 6) prepared by Gabriele Carletti which, at the time of analysis, had been naturally aged for a period of four years.

Model System	Preparation Layer Type	Preparation Layer Composition	Varnish Layer Type
1	water soluble	K_2_Cr_2_O_7_	oil based
2		K_2_Cr_2_O_7_ + NaHCO_3_	spirit-based
3		cherry gum	
4		gamboge + saffron	
5	ethyl alcohol soluble	dragon’s blood	oil based
6		sandalwood	spirit-based

## Data Availability

Not applicable.

## Sample Availability

Samples of the compound are available from the authors.
